# Interaction of Microglia and Astrocytes in the Neurovascular Unit

**DOI:** 10.3389/fimmu.2020.01024

**Published:** 2020-07-08

**Authors:** Li-rong Liu, Jia-chen Liu, Jin-shuang Bao, Qin-qin Bai, Gai-qing Wang

**Affiliations:** ^1^Shanxi Medical University, Taiyuan, China; ^2^People's Hospital of Yaodu District, Linfen, China; ^3^Xiangya Medical College, Central South University, Changsha, China; ^4^SanYa Central Hospital, The Third People's Hospital of HaiNan Province, SanYa, China

**Keywords:** microglia, astrocyte, neuroinflammation, stroke, LPS, NVU

## Abstract

The interaction between microglia and astrocytes significantly influences neuroinflammation. Microglia/astrocytes, part of the neurovascular unit (NVU), are activated by various brain insults. The local extracellular and intracellular signals determine their characteristics and switch of phenotypes. Microglia and astrocytes are activated into two polarization states: the pro-inflammatory phenotype (M1 and A1) and the anti-inflammatory phenotype (M2 and A2). During neuroinflammation, induced by stroke or lipopolysaccharides, microglia are more sensitive to pathogens, or damage; they are thus initially activated into the M1 phenotype and produce common inflammatory signals such as IL-1 and TNF-α to trigger reactive astrocytes into the A1 phenotype. These inflammatory signals can be amplified not only by the self-feedback loop of microglial activation but also by the unique anatomy structure of astrocytes. As the pathology further progresses, resulting in local environmental changes, M1-like microglia switch to the M2 phenotype, and M2 crosstalk with A2. While astrocytes communicate simultaneously with neurons and blood vessels to maintain the function of neurons and the blood–brain barrier (BBB), their subtle changes may be identified and responded by astrocytes, and possibly transferred to microglia. Although both microglia and astrocytes have different functional characteristics, they can achieve immune “optimization” through their mutual communication and cooperation in the NVU and build a cascaded immune network of amplification.

## Introduction

Neuroinflammation often runs through the entire process of pathological development. There is a dynamic change over time with the regulation of pro and anti-inflammatory signals (1, 2). Microglia/astrocytes, part of the neurovascular unit (NVU), are activated by various brain insults. The local extracellular and intracellular signals determine their characteristics and switch of phenotypes. Generally, microglia and astrocytes are activated into two states: the pro-inflammatory phenotype (M1/A1) and the anti-inflammatory phenotype (M2/A2), corresponding to either the destructive or reparative functions in the NVU, respectively (3–5). The activated microglia and astrocytes have dynamic phenotypic changes (6–9).

The crosstalk between microglia and astrocytes occurs through a variety of molecule signals such as adenosine triphosphate (ATP), cytokines, etc. (10). Liddelow et al. (9) showed that reactive astrocytes (A1) can be induced by the cytokines secreted from activated microglia (M1), which are induced by lipopolysaccharides (LPS) *in vitro* and *in vivo* (11). Microglia appear to be more sensitive to pathogens or damage, which stimulate them and promote secretion of “molecular signals” to trigger reactive astrocytes. Neuroinflammation, such as in stroke, may exhibit a similar mechanism and interaction between microglia, and astrocytes may share the common molecular language in various diseases. It has been previously shown that neuroinflammation between the microglia and astrocytes has a cascade of amplification (12–14), but its mechanism needs further elucidation. As the pathology progresses, thus causing environmental changes, it promotes the switch from M1 to M2, which is also closely associated with A2. While astrocytes, an essential component of the NVU, communicate simultaneously with both neurons and blood vessels as versatile cells to maintain the function of neurons and the blood–brain barrier (BBB), there seems to be a difference in the communication of astrocytes from microglia. This review is concerned with the origin, anatomy, and physiological function of microglia and astrocytes, particularly their communication and cooperation in pathological conditions. The activated microglia and astrocytes may achieve immune “optimization” through their interaction in the NVU.

## Microglia and Astrocytes in the NVU

The NVU, a structural and functional unit, is composed of microglia, neurons, the BBB, and the extracellular matrix (15). Its primary function is to meet the brain's dynamic metabolic needs by regulating the cerebral blood flow (CBF) in response to physiological or pathological stimuli in the CNS (16, 17). The BBB consists of vascular endothelial cells (ECs), tight junctions, and basement membranes, pericytes, or smooth muscle cells, and astrocytes. It separates parenchyma of the central nervous system (CNS) from blood, and it thus maintains a stable micro-environmental homeostasis of CNS (18, 19). The BBB maintains the low permeability through the tight-junction between sendothelial cells with membrane-bound transporters, and perivascular cells, such as pericytes, astrocytes, and the extracellular matrix, also contribute to this (17, 20). Astrocytes promote the maintenance of the BBB via sonic hedgehog and b-catenin, which strengthen the tight junction and integrity (21). Meanwhile, reactive astrocytes disrupt the local BBB by the release of vascular endothelial growth factor (VEGF), increase permeability, and allow entry of peripheral immune cells (22, 23).

Astrocytes are considered an indispensable element of the NVU or extended BBB. In the context of the NVU, astrocytes are located in the center between neurons and ECs. The strategic position of astrocytes enable them to regulate CBF to adapt to dynamic changes in neuronal metabolism and synaptic activity (18, 24). Astrocytes co-originate with neurons and oligodendrocytes and are produced in the final stages of neurogenesis (25, 26). They are the most abundant and heterogeneous glia cell type, tiling throughout the brain in a non-overlapping manner in the CNS (27).

Astrocytes are closely associated with neurons and blood vessels as versatile cells (28, 29) and communicate with neuronal pre- and post-synaptic terminals to help modulate synaptic transmission by the release of glutamate, D-serine, and ATP. It has been reported that one astrocyte can supervise over 100,000 synapses (30–33). Astrocytes can be extensively coupled into syncytial structures of up to 100 units by gap junctions, composed of connexin (CX) proteins such as CX-43 and CX−30 subtypes, allowing for the rapid facilitation of long-range signaling through calcium waves (34–37). Astrocytes extend end-feet processes to cover the surface of cerebral blood vessels with a ratio of ~99% to modulate CBF or the BBB (24). Furthermore, the end-feet with high levels of aquaporin-4 water channel proteins promote perivascular clearance by the newly characterized “glymphatic system” (CNS waste clearance system) (38, 39).

Astrocytes were, in the past, considered simply as a supportive or “glue-”like function in the CNS; now, their essential functions are increasingly being elucidated (28). Besides the above mentioned effects of “glymphatic system” (39, 40), astrocytes also have neurotrophic support, promote formation, and maintenance of synaptic activity, and transmission, regulate CBF, and determine some functions, and properties of the BBB, or NVU (27). In physiological conditions, astrocytes restrict the entry of peripheral immune cells passing through the BBB (41). While in pathological conditions, astrocytes participate in innate immune reactions (42) and the adaptive immune responses by their strategic position (43, 44).

Microglia, an important partner of the NVU, are the primary immune cells and account for ~5–15% of all cells in the human brain (45, 46). Early in development, microglia derive from the yolk sac, and seed in the brain as the first glial cells, and they develop concurrently with neurons into highly plastic cells with mobility (47–49). Under physiological or pathological conditions, microglia continuously survey their surrounding environment and always firstly respond to any insult in the CNS (50–52).

There is a local network of immune cells via communication and collaboration in the CNS against pathogenic insults, injury, or stress (44). Microglia, scattered throughout the brain, wander more observantly and detect modifications of their environment as sentinels (42, 53). Whether as the first glial cells seeded in the brain early in embryonic development or as the first to respond to insults in CNS, microglia are always the “pioneers” in the NVU. On the other hand, astrocytes with a more dominant quantity may be “reserve forces” and amplify the neuroinflammation, owing to syncytium of the structure and function and strategic position to mobilize peripheral immunity (54).

## Association of Microglia, Astrocytes, and Neuroinflammation

**Figure 1:**
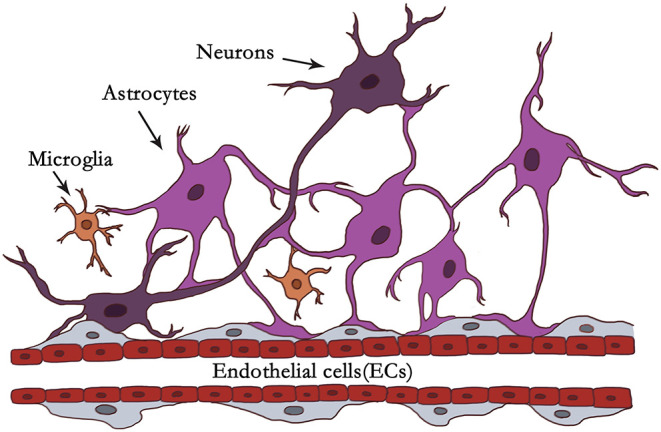
Illustration of microglia and astrocytes in NVU. In the context of the NVU, astrocytes are located in the center between neurons and endothelial cells (ECs). Astrocytes are closely associated with neurons and blood vessels as versatile cells. Astrocytes communicate with neuronal pre- and postsynaptic terminals to help modulate synaptic transmission. It has been reported that one astrocyte can supervise over 100,000 synapses. Astrocytes extend end-feet processes to cover the surface of cerebral blood vessels with a ratio of ~99% to modulate CBF or the BBB. Astrocytes can be organized into syncytial structures of up to 100 units by gap junctions to facilitate long-range signaling. Microglia account for about 5–15% of all cells in the human brain. Under physiological or pathological conditions, they scan their environment through scavenging functions. Microglia firstly react to brain insults like “pioneers,” monitoring and transmitting “danger.” Astrocytes with dominant quantity may be “reserve forces” and amplify the neuroinflammation, owing to their syncytium of the structure, and function, and strategic position to mobilize peripheral immunity.

Neuroinflammation is constantly present at every different pathological state in CNS diseases. Neuroinflammation is induced when the NVU responds to specific stimuli involved in the activation of microglia and astrocytes, breakdown of the BBB, infiltration of peripheral leukocytes, and inflammation factors, etc. (55). Activated microglia and reactive astrocytes play a crucial role in neuroinflammation. The dynamic phenotypic changes of microglia and astrocytes determine their detrimental or beneficial character at particular stages (7, 9). Microglia and astrocytes in NVU is illustrated in Figure 1.

### Microglia

Microglia, the first activated innate immune cells, can be activated within minutes of tissue damage (56). Activated microglia, with changes from the ramified morphology into an amoeboid shape, upregulate the secretion of numerous inflammation factors, and microglial phagocytosis (57). The local extracellular and intracellular signals determine their characteristics and switch of phenotypes, which range from “M1-like” phenotypes characterized by increase of inflammatory mediators, such as tumor necrosis factor (TNF), interleukin 1 beta (IL-1β), and reactive oxygen species (ROS) (58), to “M2-like” phenotypes characterized by upregulation of anti-inflammatory mediators, such as Interleukin IL-10, transforming growth factor beta (TGFβ), and glucocorticoids (59). The M1-like phenotype is considered to be destructive to NVU (60), while the M2- like phenotype is interpreted to be nerve repair cells in CNS diseases (61). Moreover, microglia display intermediate phenotypes with diverse combination of polarization markers ranging from M1 to M2, representing the crossroads of diverse pro- and anti-inflammatory (62–64). Although the supposed dichotomy of M1/M2 phenotypes hardly reflect a wide range of microglial phenotypes, this facilitates understanding of the activated state of microglia in various CNS disorders (3).

### Astrocytes

Astrocytes are another type of glial cells that actively participate in regulation of neuroinflammation, depending on the timing and context (65). Following diverse brain injuries, astrocytes undergo a significant transformation called “reactive astrocytosis,” whereby they upregulate many genes, increase the size of cytoskeleton, process extension, increase expression and immunoreactivity of glial fibrillary acidic protein (GFAP), and form a glial scar (5, 66, 67). Reactive astrocytes were purified and genetically analyzed in mice about neuroinflammation induced by systemic injection of LPS or cerebral ischemia induced by middle cerebral artery occlusion (MCAO). Neuroinflammation and ischemia induced two different types of reactive astrocytes, which correspond to “A1” pro-inflammatory and “A2” anti-inflammatory, respectively. This nomenclature is similar to the “M1” and “M2” of microglia (9). Different polarizations of astrocytes are marked by different biochemical and functional characteristics (68–70). A1 reactive astrocytes elevate levels of many genes of the classic complement cascade, such as C1r, C1s, C3, and C4, which are harmful for the NVU. Meanwhile, A2 reactive astrocytes upregulate beneficial inflammatory factors, such as CLCF1 (cardiotrophin-like cytokine factor 1), LIF (hypoxia induce factor), IL-6, IL-10, and thrombospondins, to promote the NVU remodeling (5, 9). Reactive astrocytosis also represents a spectrum of alterations reflecting the specific insults in the CNS (9, 54).

**Figure 2:**
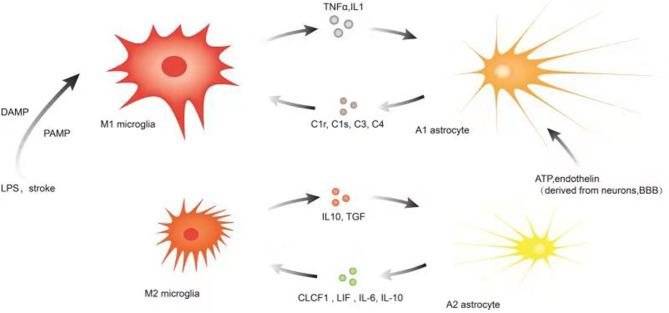
Illustration of microglia, astrocytes, and neuroinflammation. Microglia are more sensitive to pathogens/damage such as LPS or stroke, firstly activated into M1-like phenotypes via PAMP/DAMP and promote the secretion of inflammatory factors such as TNF-a, IL-1, etc. to trigger reactive astrocyte (A1). As the insult limited and the NVU is remodeling, the local environmental factors change and determine M2-like phenotype to upregulate microglial phagocytosis and secretion of IL10, TGF, etc. Simultaneously, the local environmental factors may promote the switch to A2. Astrocytes communicate simultaneously with both neurons and blood vessels as versatile cells to maintain the function of neurons and the blood–brain barrier, and their subtle changes may be captured and responded by astrocytes and even transferred to microglia. A variety of molecular signals such as ATP, endothelin, etc. trigger reactive astrocytes (A1) and A1 upregulates many genes of the classic complement cascade such as C1r, C1s, C3, and C4, which communicate with microglia via some corresponding complement receptors; A2 elevates the levels of neurotrophic factors and cytokines such as CLCF1, LIF, IL-6, IL-10, and thrombospondins to promote neuronal survival and repair; the local environmental factors promote the switch to M2-like phenotype.

The association of microglia, astrocytes, and neuroinflammation is illustrated in Figure 2.

## Interaction of Microglia and Astrocytes in the NVU

### The Common Molecular Signals of Interaction

Reactive astrocytes are induced by LPS-activated microglia (11, 56, 71). Liddelow et al. (9) showed that reactive astrocytes (A1) can be induced by cytokines, such as interleukin-1 alpha (IL-1α), TNF-α, and the complement component subunit 1q (C1q), which secreted by activated microglia (M1) both *in vitro and in vivo*. (11). Microglia appear to be more sensitive to pathogens; they activate and secrete “molecular signals” to trigger reactive astrocytes. Interaction between activated microglia and astrocytes plays a crucial role in the process of neuroinflammation. Neuroinflammation of diverse CNS diseases such as stroke may share the common “molecular signals” to trigger astrocytes reaction, and these inflammation signals may be amplified (72).

The activation of microglia occurs early in the timeline of neuroinflammation following stroke besides LPS-induced inflammation. Microglial activation within the perihematomal region, by immunofluorescence staining, was seen within 1 h of intracerebral hemorrhage (ICH) in a model of ICH (73, 74). In a clinical study of perihematomal brain tissue, TNF and IL-1β levels increased within 1 day of ICH (75, 76). After collagenase-induced or autologous blood-induced ICH, IL-1β, TNF, IL-6 (77, 78), and inducible nitric oxide synthase (iNOS) (25), mRNA levels were generally upregulated in the acute phase, starting to rise in the first 3 h after ICH and peaking at 3 days (79, 80). Changes in the protein levels corresponded to the timeline (25, 80, 81). Similarly, in the acute phase of ischemic stroke, microglia were activated first and invaded the peri-infarct and infarct core to orchestrate the post-stroke neuroinflammatory response and communicated with astrocytes through soluble and membrane-bound signaling molecules (82–84), including the cytokines IL-1β, TNF, and IL-1 receptor antagonist (IL-1Ra) (82, 83, 85). These studies imply that microglia in stroke are more sensitive to pathogens/damage; which are activated and produce then produce the common “molecular signals,” such as IL- 1 and TNF, to trigger reactive astrocytes (11, 86). Meanwhile, another study showed highly enriched astrocyte cultures produced only a very few inflammatory factors, such as TNF-α, reactive oxygen species (ROS), and nitric oxide (NO), in response to LPS stimulation. Astrocytes seem to be sluggish in response to pathogens stimulation and fail to be completely activated in the absence of microglia (87).

TNF-α is a multi-effect cytokine mostly released from microglia/macrophages (88) and neutrophils (89). The IL-1 cytokine family has a large number of members, and the most important are IL-1α, IL-1β, and the natural receptor antagonist IL-Ra (90). Further, IL-1β is mainly derived from microglia/macrophages (91). Both TNF-α and IL-1 primarily produced by microglia/macrophages are overexpressed within the first 2 h after experimental ICH (92–94) and as early as 24 h after ischemic stroke in mice (82).

Microglia are firstly activated via TLR4 by the pathogens or damage and release the inflammation mediators TNF-α (95). Sansing et al. (96) showed that activated microglia express high levels of TLR4, which result in neuroinflammation after ICH. Meanwhile, astrocytes respond through activation of TLR2, TLR3, and TLR4, almost depending on the presence of microglia (97). In the case of TLR4 activation in response to LPS, microglia directly trigger or promote astrocytic responses by upregulating the expression levels of soluble mediators. The results indicate that microglia play a critical role in astrocytic activation via TLR4 in response to insults, injury, or inflammation in CNS disorders (14, 97).

It is observed that human astrocytes are highly sensitive to IL-1β but unresponsive to LPS stimulation, and reactive astrogliosis is also induced by IL-1β alone (98). Within 24 h of IL-1β induction, large numbers of reactive astrocytes are observed, and elevate the matrixmetalloprotease (MMP)-9 expression (98–100). Although astrocytes produce certain pro-inflammatory factors, microglia are the main source of cytokines (101). Primary mediators, such as TNF, IL-1β, and IFNγ, promote the produce of secondary mediators, such as MMP, nitric oxide(NO), and arachidonic acid (72).

These evidences suggest that, in the process of neuroinflammation induced by stroke or LPS, microglia are more sensitive to pathogens/damage and activated via PAMP/DAMP and release the common “molecular signals” or primary mediators, such as IL- 1 and TNF-α, to trigger reactive astrocytes, while astrocytes are unresponsive to pathogens/damage in the absence of microglial cells.

### HMGB1

High-mobility group protein box-1(HMGB1), a highly conserved non-histone DNA-binding protein, is involved in pro-inflammatory cytokine gene transcription in diverse inflammatory diseases (56, 102–104). In a rabbit subarachnoid hemorrhage (SAH) model, the Murakami group found that the HMGB1 protein are located in microglia and macrophages with a ratio >90% (105). In a collagenase-induced mode of ICH in rats, the release of HMGB1 into the cytoplasm in the brain was detected within 1 h, and express levels of HMG1 protein was substantially elevated at 24 h after ICH (106–108). These suggest that HMGB1 also primarily arise from microglia/macrophages and seem to be produced concurrently with cytokines such as IL- 1 and TNF-α.

*In vitro*, microglia stimulated by TNF-α release large amounts of HMGB1 (109), and recombinant human HMGB1 (rhHMGB1) can activate microglia, increase NF-κB activity, and promote inflammation factors including TNF-α, IL-1β, cyclooxygenase (COX)−2, and NO (110). However, these effects disappeared in TLR4–/– microglia treated with rhHMGB1 (110). These observations indicate that not only pathogens/damage but also HMGB1 can ignite microglial activation via TLR4 and promotes the produce of TNF-α, which in turn stimulates microglia to release large amounts of HMGB1 to active more microglia. There seems to be self-feedback loop in the process of microglial activation.

### Signal Can be Amplified

Molecular languages, such as TNF-α and IL-1 is not only the proinflammatory factors of M1-like phenotypes, but more like “signals” to trigger reactivity astrocytes, and these inflammatory signals may be amplified by the unique physiological structure of astrocytes. A rat model experiment indicated that primary microglia are more sensitive to lead (Pb) exposure; compared to astrocytes, Pb is more likely to reduce microglial viability, while astrocytes have greater uptake of Pb (111). Similarly, the Kirkley group found that microglia can amplify the inflammatory activation of astrocytes by the release of cytokines and chemokines (12).

ATP and analogs interacts with G protein-coupled P2Y receptors to promote astrocyte proliferation and the growth of long, branched processes (101). It has also been shown that microglial cells quickly released small amounts of ATP, and astrocytes in turn amplified this release, increasing the frequency of excitatory postsynaptic currents through P2Y1 (14). This response can be blocked by inhibitors of connexin channels. In the case of connexin channel inhibitors, microglial movement is also significantly impeded (112). These results reveal that microglia as upstream partners ignite the response and astrocytes with the syncytium coupled by connexin channels magnify this.

In conclusion, microglia firstly react like “pioneers” in the NVU, initiate immune cascades, release inflammatory mediators, and form network regulation. Meanwhile, astrocytes with dominant quantity may be “reserve forces” and amplify the neuroinflammation, owing to their syncytium of structure and function. In addition, the amplification of neuroinflammation may be also related to astrocytic strategic position to mobilize peripheral immunity.

### Communication Between M2 and A2

As mentioned above, the M1-like microglia secrete some pro-inflammatory mediators to induce A1 astrocytes, which amplify the cascaded neuroinflammation. With the process of the insults limited and the NVU remodeling, the local environmental factors change and determine the switch of microglial and astrocytic phenotypes. Activated microglia-Derived Cytokines (TNF-α, IL-1β and IL-6) induced the switch of astrocyte phenotype after brain trauma (113). The interaction of microglia and astrocytes plays a vital role in the switch of phenotypes. In addition, activated M2-like microglia produce the anti-inflammatory cytokine IL-10, which matches the IL-10 receptor (IL-10R) primarily expressed in A2 astrocytes, and this allows astrocytes to secrete TGF-β, which reduces microglial activation (114). The communication between M2 and A2 significantly promotes neuronal survival and repair and is even amplified by the unique anatomy structure of astrocytes.

### Astrocytic Dialogue to Microglia

In physiological conditions, astrocytes communicate simultaneously with both neurons and blood vessels as versatile cells to maintain the function of neurons and the blood–brain barrier (27). In pathological conditions, reactive astrogliosis, and astrocytic proliferation become dominant, and the process is triggered by diverse molecular signals, such as cytokines, ATP, endothelin, sonic hedgehog, fibroblast growth factor2 (FGF2), thrombin, and bone morphogenic proteins (BMP) (27, 115). The communications among neurons, BBB and microglia/macrophage mostly rely on these molecular signals (11, 13, 21, 116). Early triggers contain nucleotides released from damaged cells and pro-inflammatory cytokines as well as purines/pyrimidines such as ATP and elevated excitotoxic transmission, as ATP is co-released with neurotransmitters (101). In physiological or pathological conditions, astrocytes seem to primarily sense the signals derived from neurons and blood–brain barrier components including microglia in the NVU.

During development, astrocytes can sense subtle changes in neurons to induce the production of C1q in neuronal synapses, which interacts with the microglial C3a receptor (C3aR) to prune the neuronal synapses through the classic cascade complement pathway (117). In the context of Alzheimer's disease (AD) pathology, overproduction of AD promote the release of C3 from astrocytes, which simultaneously communicate with microglial C3aR and neuronal C3aR to dynamically regulate microglial phagocytosis and impair dendritic morphology as well as synaptic function, subsequently deteriorate cognitive function. The damaged neurons in turn trigger more astrocytes and active more microglia. Complement-dependent intercellular crosstalk is critical to promote the pathogenic cycle, and the feedforward loop can be blocked effectively by C3aR inhibition (118, 119).

Astrocytes are major sources of many chemokines, such as CCL2, CXCL1, CXCL10, and CXCL12 (120–122), and microglia express some corresponding chemokine receptors, such as CCL2, CXCL12 (123, 124), and so on. This implicates a strong association between microglia and astrocytes.

In summary, astrocytes, one of the important components of the NVU, communicate simultaneously with both neurons and blood vessels as versatile cells to maintain the function of neurons and the blood–brain barrier, whose subtle changes are captured and responded to by astrocytes, and even transferred to microglia. In early mild cognitive impairment, astrocytes may be the primary responsibility for this, but, in moderate or severe cognitive impairment such as AD, amounts of accompanied neurons death or apoptosis may also directly activate microglia, as microglia are more sensitive to pathogens/damage and trigger more reactive astrocytes via inflammatory signals, which can be amplified not only by the self-feedback loop of HMGB1 but also by the unique anatomy structure of astrocytes. Although both microglia and astrocytes own their functional characteristics, they can achieve the immune “optimization” through their mutual communication and cooperation in neuroinflammation.

**Figure 3:**
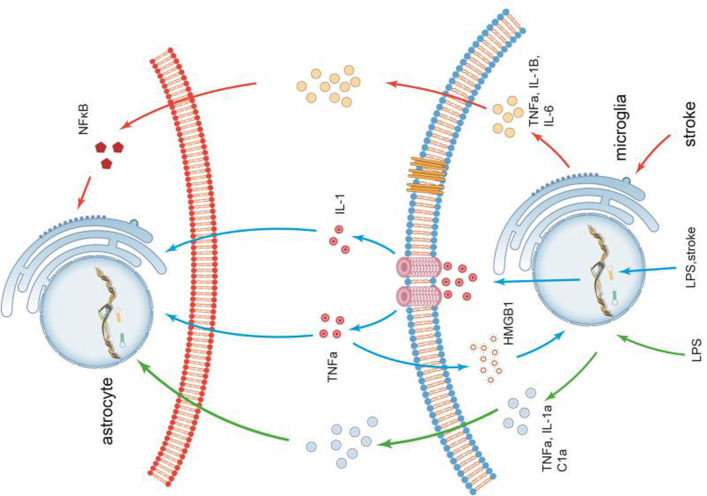
The communications of microglia and astrocytes. Pathogens/damage trigger M1-like microglia via TLR4. During the neuroinflammation induced by LPS or stroke, Microglia are more sensitive to pathogens/damage, firstly activated, and secrete the common “molecular signals,” such as IL-1 and TNF-a, to trigger reactive astrocytes. Different types of insults release different combinations of these molecules, which in turn trigger different responses. It has been demonstrated that inflammation factors induced by LPS, such as TNF-α, IL-1a, and C1a, can trigger reactive astrocytes. In stroke, however, the inflammatory factors secreted by activated microglia(M1), such as TNF-α, IL-1β, and IL-6, are significantly elevated. Recombinant human HMGB1 (rhHMGB1) can trigger microglial activation via the TLR4 and increase production of TNF-α, which in turn stimulates microglia to release large amounts of HMGB1 to active more microglia. There seems to be self-feedback loop in the activation process of microglia.

The communications of microglia and astrocytes is illustrated in Figure 3.

### Different Pathogens/Damage and Different Effects

The inflammatory effects of the central nervous system depend on several parameters, including the types and severity of pathogens/damage, glial cell types, a variety of combinations of signal molecules (including chemokines, cytokines, etc.), and timeline of the response, etc. (112, 125, 126).

Zamanian et al. showed that reactive astrocytes induced by LPS or ischemic stroke upregulate over 1,000 genes, and genomic profiling has shown that both gene representation and fold induction correspond to individual injuries. Some of the upregulated genes are unique to the LPS subtype (A1) or the middle cerebral artery occlusion (MCAO) subtype (A2). For example, the three genes, including *Ptx3, S1Pr3*, and *tweak*, are markers for the MCAO subtype (A2), while *H2-D1* and *Serping1* are markers for the LPS subtype (A1) of reactive astrocytes. *H2-D1* was induced 30-fold by LPS but only 3-fold by MCAO. *Serping1* was induced 6.5-fold after MCAO and 34-fold after LPS (5). These data indicate that different pathogens/damage can induce different phenotypes of astrocytes. This may closely relate to the different interaction between the activated astrocytes and microglia, which contain different combinations of molecule signals. It has been demonstrated that inflammation factors induced by LPS, such as TNF-α, IL-1a, and C1al, can trigger reactive astrocytes (11). In stroke, the inflammatory factors secreted by activated microglia (M1), such as TNF-α, IL-1β, and IL-6, are significantly elevated (77, 78, 127). Furthermore, a recent clinical inflammatory factor test is about the relationship of inflammatory markers and severity of ICH, and this test displayed that high TNF-a is closely associated with the size of edema around the hematoma and increase of early hematoma, leading to poor functional recovery and high mortality (128). These studies imply the different types and severities of insults release different combinations or levels of these molecule signals, which in turn trigger different responses.

Thus, different pathogens/damage correspond to different phenotypes of glia cells. Even the same pathogens/damage with the different levels of stimulation, the activated levels and phenotypic timeline of glia cell are also different. In the pathology of neuroinflammation induced by LPS, the pathogen is stronger, M1/A1 are primary, and it is critical to suppress the pro-inflammatory or shorten the phase. While sterile inflammation induced by stroke, such as cerebral infarction or hemorrhage, it may be beneficial to moderately attenuate the activation levels and shorten the timeline of M1/A1 or strengthen A2/M2, some studies and experiments have confirmed this (85, 129–131). In degenerative disease, such as AD, it may be beneficial to enhance the A2/M2 for brain repair and functional recovery. While in autoimmune diseases such as multiple sclerosis, autoimmune encephalitis, attenuating M1/A1 in time may be more beneficial (132–139).

## Summary and Outlook

Neuroinflammation is dynamic with the regulation of pro and anti-inflammatory signals. The activation and interaction of glial cells play a crucial role at different stages of pathology in CNS disorders. Microglia are more sensitive to pathogens or damage, firstly activated (M1) like “pioneers,” monitoring and transmitting “danger” via the common molecule signals to trigger reactive astrocytes (A1). Astrocytes with dominant quantity may be “reserve forces” and amplify the neuroinflammation, owing to their syncytium of the structure, and function, and strategic position to mobilize peripheral immunity. Although inflammation signals between microglia and astrocytes may share the common inflammatory signals, such as IL- 1 and TNF-α, different pathogens and pathological conditions may correspond to different inflammatory signals (TNF-α, IL-1β, and IL-6 are significantly elevated in stroke, while TNF-α, IL-1a, and C1a induced by LPS can trigger markedly reactive astrocytes), and different strategies may be required. As the pathology further progresses, the communication between M2 and A2 significantly promote neuronal survival and repair. In addition, astrocytes, as one of the essential components of the neurovascular unit (NVU), communicate simultaneously with both neurons and blood vessels as versatile cells to maintain the function of neurons and the BBB; their subtle changes are identified and responded by astrocytes and even transferred to microglia. Activated microglia and reactive astrocyte may achieve the immune “optimization” through mutual communication and cooperation in neuroinflammation. Inflammation signals between microglia and astrocytes can be amplified not only by the self-feedback loop of microglial activation but also by the unique anatomy structure of astrocytes in the immune network (140–148). With advancements in technology, the interaction of microglia and astrocytes may be an effective and accurate therapeutic target in the future.

## Author Contributions

GW revised the work critically for intellectual content and contributed equally to the work. JL contributed to the drawing. JB and QB assisted drawing. All authors contributed to the article and approved the submitted version.

## Conflict of Interest

The authors declare that the research was conducted in the absence of any commercial or financial relationships that could be construed as a potential conflict of interest.

## Abbreviations

NVU: Neurovascular unit
LPS: Lipopolysaccharides
CNS: Central nervous system
ATP: Adenosine triphosphate
BBB: Blood–brain barrier
ECs: Endothelial cells
VEGF: Vascular endothelial growth factor
MMP-9: Matrix metalloproteinase-9
CX: Connexin
CBF: Cerebral blood flow
ROS: Reactive oxygen species
TNF: Tumor necrosis factor
IL-1β: Interleukin 1 beta
IL-1α: Interleukin-1 alpha
iNOS: Inducible nitric oxide synthase
TGFβ: Transform growth factor beta
CLCF1: Cardiotrophin-like cytokine factor 1
LIF: Hypoxia induce factor
C1q: Complement component subunit 1q
SAH: Subarachnoid hemorrhage
PAMP: Pathogens associated molecular pattern
DAMP: Damage associated molecular pattern
HMGB1: High-mobility group protein box-1
COX: Cyclooxygenase
Pb: Plumbum
GFAP: Glial fifibrillary acidic protein
AD: Alzheimer's disease
NO: Nitric oxide
MCAO: Middle cerebral artery occlusion.
